# Supplementary material for: Long-Term Salinity-Responsive Transcriptome in Advanced Breeding Lines of Tomato
Source: Plants (Basel). 2025 Jan 1;14(1):100. doi: 10.3390/plants14010100 (PMC11722587; doi:10.3390/plants14010100)
Supplement: Supplementary file 1 [file plants-14-00100-s001.zip › plants-3360501-supplementary.pdf]

Supplementary Table S1: SOS genes in tomato and orthologs in Arabidopsis.

Gene	Arabidopsis	Tomato	
SOS1	AT2G01980	Solyc01g005020	Other names: ARABIDOPSIS NA+/H+ ANTIPORTER 7, ARABIDOPSIS SALT OVERLY SENSITIVE 1, ATNHX7, ATSOS1, SALT OVERLY SENSITIVE 1, SOS1<br>Encodes a plasma membrane-localized Na+/H+ antiporter SOS1. Functions in the extrusion of toxic Na+ from cells and is essential for plant salt tolerance. Has 12 predicted transmembrane domains in the N-terminal region and a long cytoplasmic tail of approx. 700 aa at the C-terminal side. SOS1 interacts through its predicted cytoplasmic tail with RCD1, a regulator of oxidative-stress responses, suggesting that SOS1 might function in oxidative-stress tolerance.
SOS2	AT5G35410	Solyc12g009570	Other names: ATSOS2, CBL-INTERACTING PROTEIN KINASE 24, CIPK24, SALT OVERLY SENSITIVE 2, SNF1-RELATED PROTEIN KINASE 3.11, SNRK3.11, SOS2encodes a member of the CBL-interacting protein kinase family, is a regulatory component controlling plant potassium nutrition
SOS3	AT5G24270	Solyc08g077770	Other names: ATSOS3, CALCINEURIN B-LIKE PROTEIN 4, CBL4, SALT OVERLY SENSITIVE 3, SOS3<br>encodes a calcium sensor that is essential for K+ nutrition, K+/Na+ selectivity, and salt tolerance. The protein is similar to calcineurin B. Lines carrying recessive mutations are hypersensitive to Na+ and Li+ stresses and is unable to grow in low K+. The growth defect is rescued by extracellular calcium.
SOS4	AT5G37850	Solyc02g091340	Other names: ATSOS4, SALT OVERLY SENSITIVE 4, SNO1, SOS4<br>Encodes a pyridoxal kinase required for root hair development. Mutants are hypersensitive to Na+, K+ and Li+.
SOS5	AT3G46550	Solyc03g112880	Other names: FASCICLIN-LIKE ARABINOGALACTAN-PROTEIN 4, FLA4, SALT OVERLY SENSITIVE 5, SOS5<br>Isolated in a screen for salt hypersensitive mutants. Mutants have thinner cell walls, abnormal siliques and root growth is inhibited under salt stress. The gene has similarity to arabinogalactan proteins and domains associated with cell adhesion.SOS5 is required for normal mucilage adherence to seeds.
SOS6	AT1G02730	Solyc09g075550	Other names: ATCSLD5, CELLULOSE SYNTHASE LIKE D5, CELLULOSE SYNTHASE-LIKE D5, CSLD5, SALT OVERLY SENSITIVE 6, SOS6<br>Encodes a gene similar to cellulose synthase. Knock-out mutant has reduced growth, reduced xylan level and reduced xylan synthase activity in stems.It's expression is cell cycle dependent and it appears to function in cell plate formation.

Supplementary Table S2: Gene description and fold change for top 30 DEGs in L56 under salinity compared to control.

Feature ID	Fold	Description
Solyc03g116890.2.1	195.651	WRKY transcription factor 2
Solyc10g075150.1.1	187.073	Non-specific lipid-transfer protein
Solyc00g174340.1.1	173.649	Pathogenesis-related protein 1b
Solyc06g030470.2.1	134.735	Auxin-regulated protein
Solyc03g093080.2.1	124.267	Xyloglucan endotransglucosylase/hydrolase 9
Solyc08g016210.2.1	114.604	LRR receptor-like serine/threonine-protein kinase, RLP
Solyc10g045240.1.1	92.712	Beta-glucosidase D4
Solyc03g093120.2.1	89.295	Xyloglucan endotransglucosylase/hydrolase 9
Solyc04g074680.1.1	87.309	Unknown Protein
Solyc07g049530.2.1	71.967	1-aminocyclopropane-1-carboxylate oxidase
Solyc09g011860.2.1	65.494	Os03g0169000 protein (Fragment)
Solyc03g115930.1.1	63.321	Calmodulin-like protein
Solyc04g079400.2.1	60.481	Protein serine/threonine kinase
Solyc03g083730.1.1	52.102	Pectinesterase
Solyc04g079360.1.1	51.371	MYB transcription factor
Solyc01g010060.2.1	46.79	Unknown Protein
Solyc08g068610.2.1	45.899	Decarboxylase family protein
Solyc03g026230.1.1	43.927	Multidrug resistance protein mdtK
Solyc04g077270.2.1	38.097	Serine/threonine kinase receptor
Solyc03g122350.2.1	38.055	Cytochrome P450
Solyc03g094160.2.1	37.827	Metacaspase
Solyc03g093110.2.1	36.963	Xyloglucan endotransglucosylase/hydrolase 9
Solyc04g074000.2.1	34.978	Receptor like kinase, RLK
Solyc01g105450.2.1	32.973	ABC transporter G family member 11
Solyc05g050560.1.1	32.96	Transcription factor
Solyc07g005100.2.1	28.192	Chitinase-like protein
Solyc06g009110.2.1	28.126	Nucellin-like aspartic protease (Fragment)
Solyc04g025650.2.1	27.386	Monooxygenase FAD-binding
Solyc07g049660.2.1	27.206	Acetyl coenzyme A cis-3-hexen-1-ol acetyl transferase
Solyc11g068620.1.1	27.164	NAC-domain protein

Supplementary Table S3: Sequence of primers for selected salt stress responsive genes in tomato.

#	Gene description	Gene locus	Primer Code	Sequence 5′–3′ (Forward and reverse)	Tm	Size (bp)
1	Calcium-dependent protein kinase	Solyc03g031670.2.1	1101_F	CATAGACATCGGCTAGAAAGGGATGAG	59.5	157
			1101_R	CCTCTGCGATTATCTCCTTGATGG	58.7	
2	Receptor like kinase, RLK	Solyc02g091840.2.1	1108_F	CTAAGTTCTTGCAAGATTCAGGGACATC	58.8	155
			1108_R	TGCCACTTACCAGTTCTAGTAGCACC	59.3	
3	DEAD-box ATP-dependent RNA helicase 5	Solyc11g007610.1.1	1115_F	CACCAGATTCAGGGCCTGAAGAAC	59.8	165
			1115_R	GGATTCAACCGCATCAACATAACC	58.3	
4	NAC-domain protein	Solyc11g068620.1.1	1116_F	TCAACTCTTGCTATTCCAAAGTTAAGGC	58.6	179
			1116_R	GCCTCCATTTTAGTGACATTTTCCATAG	58.6	
5	Glycoside hydrolase family 28 protein / polygalacturonase family protein	Solyc05g049980.2.1	1123_F	GTAGTGGCTGAGAACGTGTCAATGG	58.7	164
			1123_R	GGCTGCACACCACTAGTAATTCCTTC	58.4	
6	Pathogenesis-related protein 1a	Solyc01g106620.2.1	1129_F	CCACCAGGTAACTGGAGAGGACAAC	58.9	180
			1129_R	CCCAAGTCACATAAGCATAGCCTGG	59.8	
7	Solute carrier family 35 member F4	Solyc09g061320.2.1	1131_F	CAACTTTGCTAACCTTGAGCCATTTAAC	58.8	179
			1131_R	CAATTGCTGCTAGAGGAACCTGAATG	59.3	
8	Glutathione peroxidase	Solyc06g073460.2.1	1137_F	TTGTGGACAAAGAAGGAAAGGTTGTG	59.4	165
			1137_R	GATAGAACCATGAAAGCACTTCCAACTG	59.4	
9	Cytochrome P450	Solyc08g005610.2.1	1142_F	GTCACCAAAACCCAATACATTCATGC	59.3	165
			1142_R	GCAAATGGCCCATACTGAATTCC	58.3	
10	Actin		1151_F	AGGATCCATCCTTGCATCACTTAGC	58.7	166
			1151_R	TAATTGCCCTTCTTTCATAGCCCC	58.6	

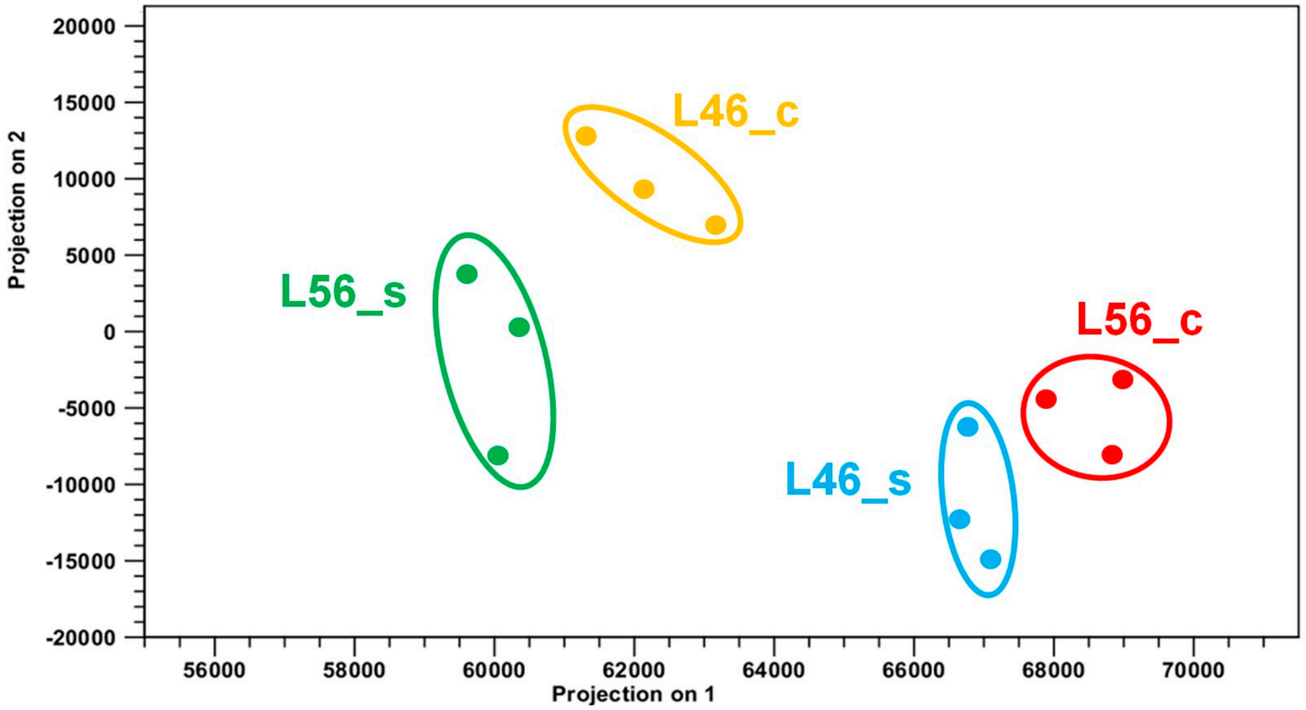

Supplementary Fig. S1 PCA for the biological replicates of RNA-seq data in tomato susceptible (L46) and tolerant (L56) genotypes under control (_c) or under salinity stress (_s) conditions.

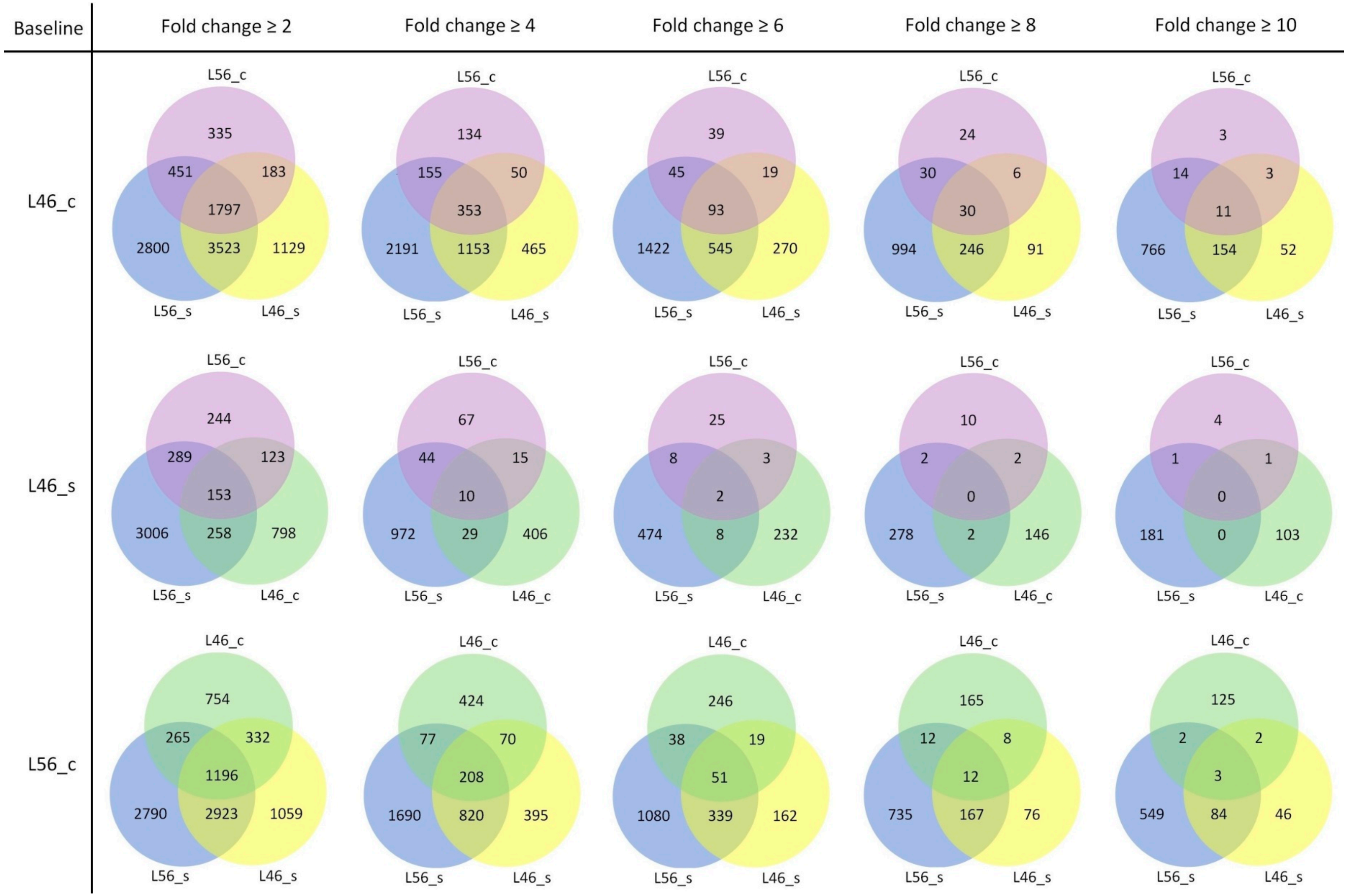

Supplementary Fig. S2 Venn diagrams showing number of overlapping up-regulated genes expressed in tomato susceptible (L46) and tolerant (L56) genotypes under control (_c) or under salinity stress (_s) conditions. The fold changes are calculated in reference to the “baseline (left column).

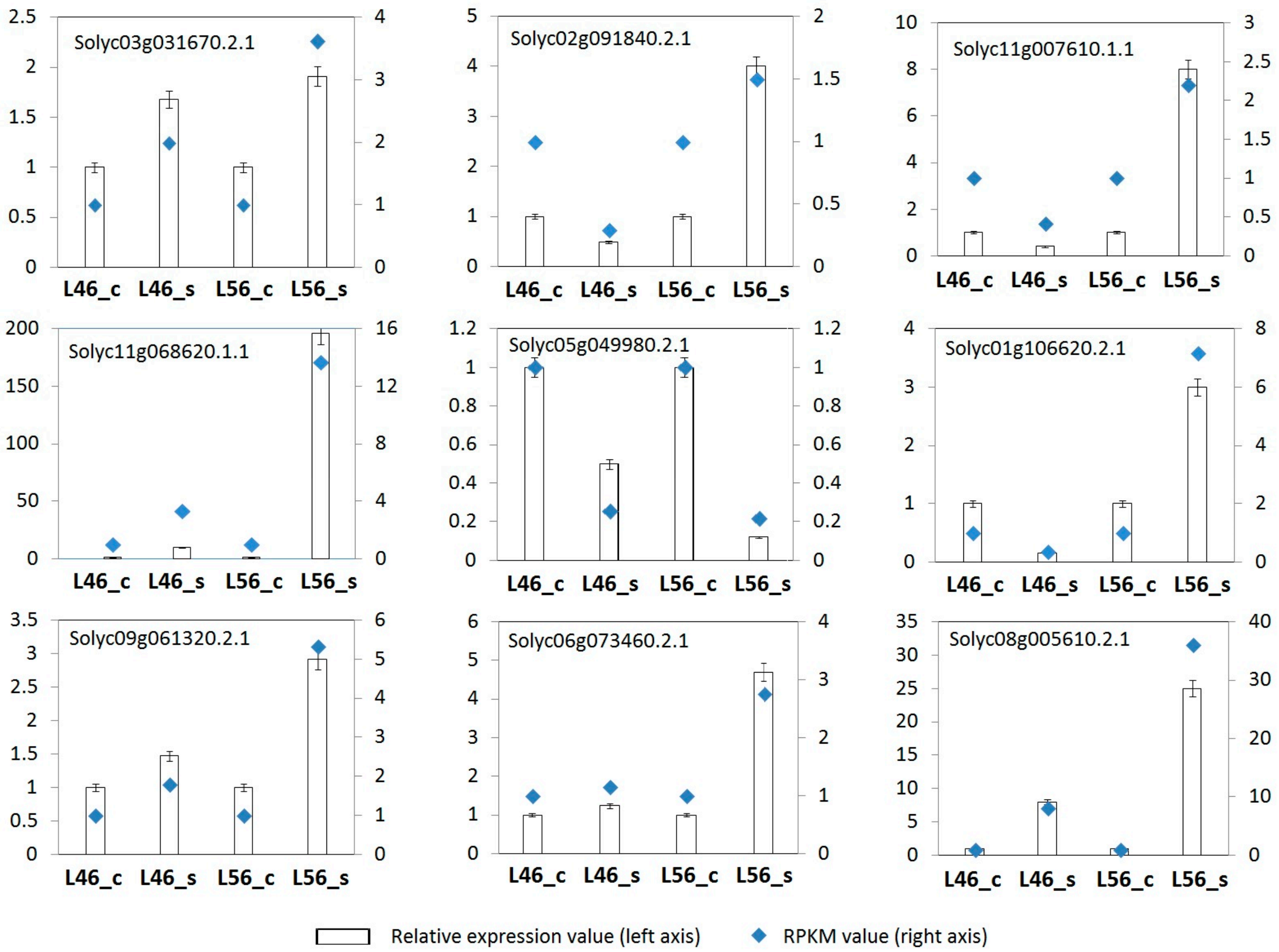

Supplementary Fig. S3 RNA-seq validation by quantitative real-time PCR (qRT-PCR). Each graph (=locus) contains the relative expression level of the two tomato genotypes; susceptible (L46) and tolerant (L56) under control (_c) or under salinity stress (_s). Bars represent data from qRT-PCR, while lines indicate data from RNA-seq. Gene description is available above (Supplementary Table S2).

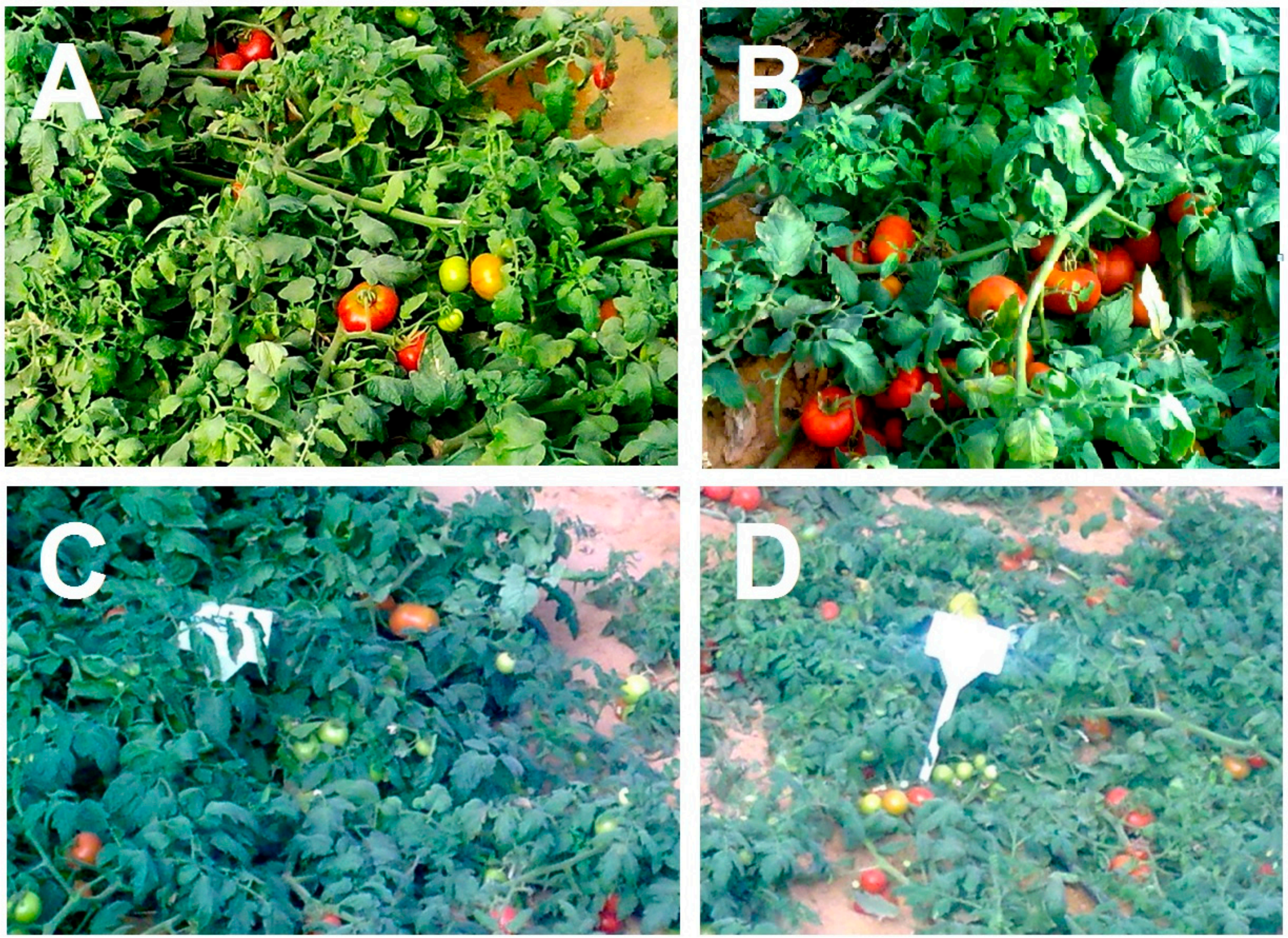

Supplementary Fig. S4 Tomato plants under control conditions (A) L56 and (B) L46 and under salinity stress conditions (C) L56 and (D) L46..
